# Potential role of myeloid-derived suppressor cells in transition from reaction to repair phase of bone healing process

**DOI:** 10.7150/ijms.51946

**Published:** 2021-02-19

**Authors:** Hotaka Kawai, May Wathone Oo, Hidetsugu Tsujigiwa, Keisuke Nakano, Kiyofumi Takabatake, Shintaro Sukegawa, Hitoshi Nagatsuka

**Affiliations:** 1 Department of Oral Pathology and Medicine, Graduate School of Medicine, Dentistry and Pharmaceutical Sciences, Okayama University, Okayama, Japan.; 2 Department of Life Science, Faculty of Science, Okayama University of Science, Okayama, Japan.; 3 Department of Oral and Maxillofacial Surgery, Kagawa Prefectural Central Hospital, Takamatsu, Kagawa 760-8557, Japan.

**Keywords:** myeloid-derived suppressor cells (MDSC), bone healing, transition period, new bone formation

## Abstract

Myeloid-derived suppressor cells (MDSCs) are a heterogeneous population of immature myeloid cells with immunosuppressive functions; these cells play a key role in infection, immunization, chronic inflammation, and cancer. Recent studies have reported that immunosuppression plays an important role in the healing process of tissues and that Treg play an important role in fracture healing. MDSCs suppress active T cell proliferation and reduce the severity of arthritis in mice and humans. Together, these findings suggest that MDSCs play a role in bone biotransformation. In the present study, we examined the role of MDSCs in the bone healing process by creating a bone injury at the tibial epiphysis in mice. MDSCs were identified by CD11b and GR1 immunohistochemistry and their role in new bone formation was observed by detection of Runx2 and osteocalcin expression. Significant numbers of MDSCs were observed in transitional areas from the reactionary to repair stages. Interestingly, MDSCs exhibited Runx2 and osteocalcin expression in the transitional area but not in the reactionary area. And at the same area, cllagene-1 and ALP expression level increased in osteoblast progenitor cells. These data is suggesting that MDSCs emerge to suppress inflammation and support new bone formation. Here, we report, for the first time (to our knowledge), the role of MDSCs in the initiation of bone formation. MDSC appeared at the transition from inflammation to bone making and regulates bone healing by suppressing inflammation.

## Introduction

Myeloid-derived suppressor cells (MDSCs) are a heterogeneous population of immature myeloid cells that play a distinct role in immunosuppression. There are two major subpopulations of MDSCs, monocytic MDSCs (M-MDSCs) and polymorphonuclear or granulocytic MDSCs (PMN-MDSCs); phenotypically and morphologically, these subpopulations are akin to monocytes and neutrophils, respectively 1,2. Recently, fibrocytic MDSCs (F-MDSCs) were characterized in humans as circulating fibrocytes with T cell-mediated immune suppressive functions 3. In mice, total MDSCs have been characterized as GR1- and CD11b-expressing cells. MDSCs have been described primarily in the bone marrow, peripheral blood, spleen, liver, lung, or tumors of various organs. MDSCs are not detectable under physiological conditions and emerge only in pathological conditions such as infection, immunization, chronic inflammation, and cancer 4-8. MDSCs are known for their role in immune suppression in cancer, and for their functions in facilitating tumor development and metastasis 9-13. MDSCs also function as osteoclast progenitors and contribute to cancer-induced osteolysis 14,15. MDSCs play a crucial role in regulating mouse collagen-induced arthritis and rheumatoid arthritis by inhibiting the proinflammatory immune response 16. However, MDSC involvement in the bone healing process is unknown.

The healing process of bone is a physiologically complicated process involving multicellular interactions that comprise three major phases: reaction, repair, and remodeling 17. Transition from reaction to repair is critical. Initial inflammation is important for the successful healing process. However, excess inflammation results in failure of the healing process 18,19. Counterbalancing of inflammation is indispensable for bone healing. Notably, the balance of effector T cell and regulatory T cell (Treg) has been reported to play a key role in bone healing 20. Elevation of the number of Treg cells improves bone healing. Moreover, in human and mouse, intermittent parathyroid hormone-induced Treg cell proliferation has been shown to provide promising results in osteoblast proliferation and bone formation 21. MDSCs impart immunosuppressive functions by stimulating the proliferation of Treg cells. Thus, we speculated that MDSCs might play a role in the bone healing process. In the present study, we created bone injury in mouse and investigated the emergence of MDSCs during the bone healing process. Our results demonstrated that MDSCs occur only transiently in the bone healing process, but these cells have the potential to play a role in bone healing by emerging in the transitional period and initiating the repair phase.

## Materials and Methods

### Experimental animals

A total of 12 female mice (C57BL/6) were purchased from Charles River Laboratories Japan, Inc. Mice were housed under pathogen-free conditions. This research was approved by the Animal Experiment Control Committee of Okayama University, Graduate School of Medicine, Dentistry and Pharmaceutical Sciences (Approval No. 05-006-099). All mouse experiments were conducted in accordance with procedures approved by the Okayama University “Guidelines for the Care and Use of Laboratory Animals”.

### Bone Injury model

**Figure 1:**
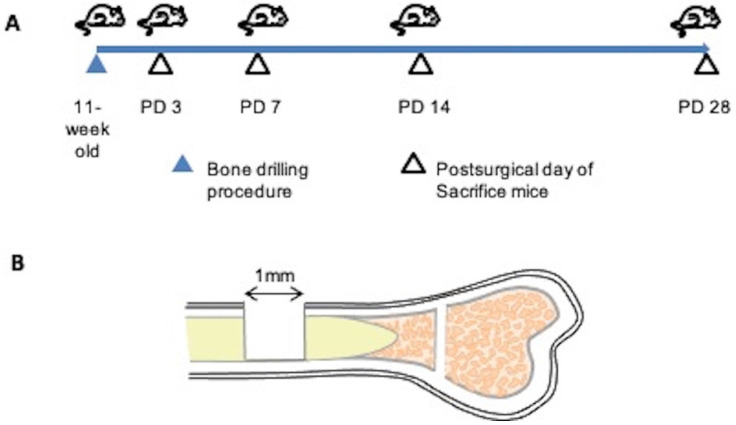
Bone injury model. (A) Demonstration of experimental procedure. Bone injuries were created in 11-week old C57BL/6 wild-type mice. Mice of separate groups (n=3 each) were euthanized at post-surgical day (PD) 3, 7, 14, or 28; the tibias were recovered at necropsy and subjected to histopathological assessment. (B) Illustration of the bone injury site. A bone injury consisting of a 1-mm-diameter hole was generated at the center of the tibial bone cortex, approximately 5 mm from the epiphysis.

A skeletal injury model was generated as described by Kim et al. 22. We used a dental laboratory MARATHON Micromotor N3 35000 RPM instrument equipped with a BUSH steel bar (round, 1.0 mm, Catalog 4290008) for generating drill holes. All procedures were performed under general anesthesia. Using a 1.0-mm drill bit, a 1.0-mm-diameter hole was created in the center of the tibial bone cortex, approximately 5 mm from the epiphysis. Mice of separate groups (n=3 each) were euthanized at post-surgical day (PD) 3, 7, 14, or 28, and tibias were collected (Figure 1).

### Histological examination

Collected mouse tibias were fixed in 4% paraformaldehyde for 12 h and decalcified in 10% EDTA at 4 °C for 14 days. Samples then were sequentially dehydrated in 70% ethanol and embedded in paraffin. Serial sections (5-μm thicknesses) were prepared. Sections were subjected to hematoxylin and eosin (HE) staining using standard methods, and to immunohistochemistry (IHC) and double IHC staining as described below.

### Immunohistochemistry

Paraffin-embedded tissue sections were deparaffinized in a series of xylene solutions for 15 min, rehydrated in graded ethanol solutions, and incubated in a solution of 3% hydrogen peroxide in methanol for 30 min to quench endogenous peroxidases. Antigen retrieval then was performed, with the technique employed depending on the respective antibody. Details of the primary antibodies used and the antigen retrieval methods are shown in Table 1. Following antigen retrieval, sections were treated with 10% normal serum (Vector Lab, Burlingame, CA) for 30 min at room temperature in a humidified chamber, followed by incubation with primary antibodies at 4 °C overnight. Secondary biotinylated antibody was applied using the avidin-biotin complex method (Vector Lab, Burlingame, CA). Color development was performed with 3, 3'-diaminobenzidine (DAB) (Histofine DAB substrate; Nichirei, Tokyo, Japan) and counterstained with Mayer's hematoxylin. Staining results were evaluated using an optical microscope.

Double IHC staining was performed using anti-GR1 and anti-CD11b antibodies. Binding by the first primary antibody (anti-GR1) was detected as described above and color development was performed using DAB liquid. In a second round of staining, sections were further stained to detect a different antigen by incubation with a second primary antibody (anti-CD11b) for 1 h, followed by its corresponding secondary antibody (Vector Lab, Burlingame, CA). For the second-round staining, color development was performed using 3-amino-9-ethyl carbazole (AEC) (Dako Cytomation). Counterstaining then was performed with Mayer's hematoxylin.

### Statistical analysis

The number of positively labeled cells was counted manually in fields at 400× (n = 3 fields). Data are presented as mean ± standard deviation (s.d.) where appropriate, and were analyzed using two-tailed non-paired Student's t-tests by using excel software. Differences were considered significant at P < 0.05.

## Results

### Bone injury models

To evaluate the bone healing process, we first created a mouse model of bone injury. The injury was generated in the tibias of female C57BL/6 mice, and sequential evaluations were performed at PD 3, 7, 14, and 28.

**Figure 2:**
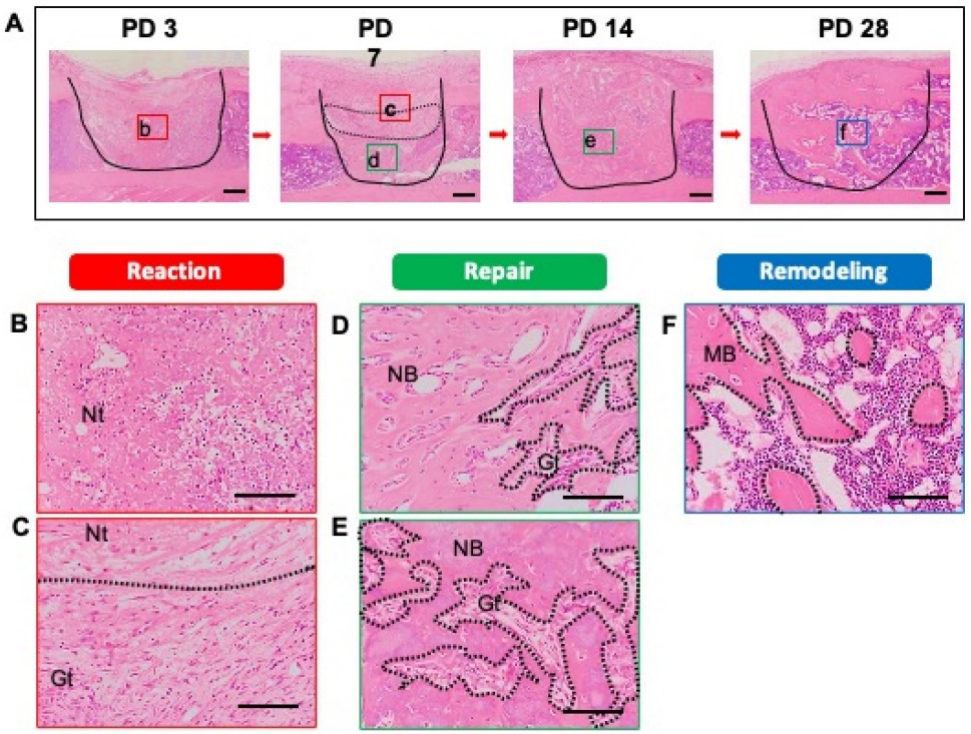
Histological evaluation of bone healing process. To detect the phases of the bone healing process, a bone injury was created in the mouse tibia, and hematoxylin and eosin staining was performed at PD 3, 7, 14, and 28 (n = 3 mice for time point). (A) Injury site is demarcated by the solid line. (B) Early reaction to the injury (hematoma formation, necrotic tissue, and inflammatory cell infiltration) was observed at PD 3. (C, D) At PD 7, the injury site showed reaction and repair within a transitional area: above the transitional area (surrounded by the dotted line), the tissue was in the early reaction phase; below, the tissue is in the repair phase. Remaining necrotic tissue, granulation tissue, and new bone formation also were observed. (E) At PD 14, the injury site had entered the repair phase. (F) At PD 28, the injury site was undergoing remodeling, and showed mature bony tissue formation and bone marrow restoration. Nt: necrotic tissue, Gt: granulation tissue, NB: new bone, MB: mature bone. Scale bars: A, 200 µm; B, C, D, E, and F, 100 µm. Lower-case letters b-f indicate regions in micrographs in A that are provided (in expanded form) in the corresponding upper-case panels B to F.

As a first step, we identified the stages of the bone healing process by HE staining of samples of injured tibias recovered at PD 3, 7, 14, and 28. All stages of the healing process (reaction (early and late), repair, and remodeling) were observed. At PD 3, early reaction to the injury, such as hematoma formation and necrotic bone tissue, was observed and there was infiltration of some inflammatory cells into the injury site (Figure 2A). The infiltrating inflammatory cells consisted of neutrophils and macrophages (Figure 2B). At PD 7, the tissue at the injury site not only showed a reaction (early and late), but also entered the repair phase of the healing process (Figure 2A). Notably, a portion of the blood clot and necrotic bone remained immediately beneath the thickening periosteum, and late reaction to injury (e.g., granulation tissue with newly formed blood vessels) was observed. The inflammatory cell infiltration persisted (Figure 2C). At the base of the injury, tissue was undergoing the repair process and showed the formation of bony callus (Figure 2D). The newly formed bony callus was surrounded by granulation tissue, indicating that the granulation area (late reaction) comprised the transitional area from the reaction to repair stages. At PD 14, an immature bony structure fully occupied the injury site and intact periosteum covered the bony injury (Figure 2A). Inflammation had subsided and granulation tissue was in close proximity to the newly formed bony tissue (Figure 2E). At PD 28, the bony continuity and marrow space were restored. Mineralized osteoid with osteocytes was observed (Figure 2A, F).

### Tissue at PD 7 shows significant accumulation of GR1- and CD11b-positive cells

**Figure 3:**
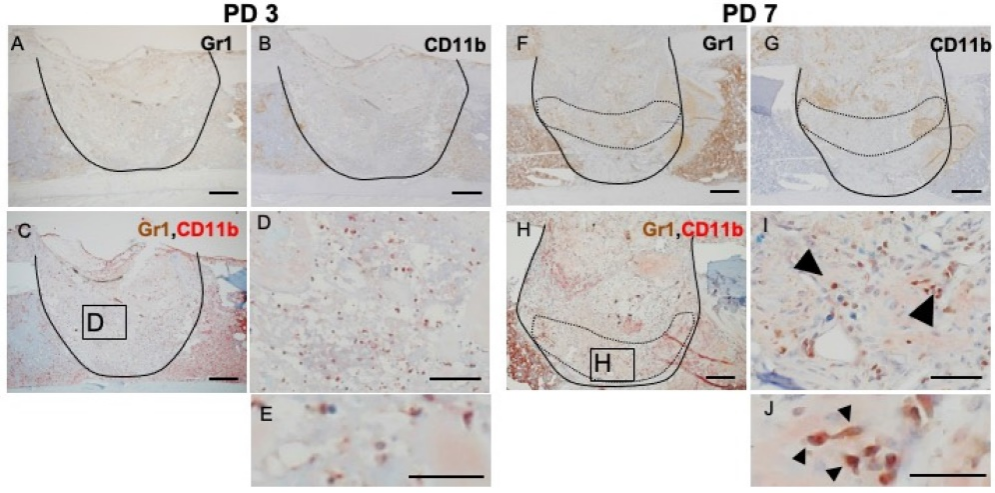
IHC and double IHC characterization and localization of Gr1 and CD11b expression in bony injury site. Gr1-positive cells were rounded and spindle-shaped. **At PD3**, Gr1- and CD11b-positive cells were widely distributed at the injury site (A, B). IHC double staining revealed the presence of low numbers of double-positive cells (C, D, E). **At PD7**, however, Gr1 expression localized specifically in the transition area (surrounded by the dotted line) (F). CD11b-positive cells were still present in the injury site, particularly at the inflammatory area (above the area surrounded by the dotted line) (G). Interestingly, double-positive cells (*arrowheads*) were gathered in the granulation area proximal to the repair area (H, I, J). Scale bars: A, B, C, F, G, and H, 200 µm; D, E, I, and J, 100 µm.

Inflammation is a naturally occurring body defense mechanism and is essential for the healing process. However, the balance of inflammation also is important for entering the repair stage. This fact made us interested in the role of MDSCs in the healing process. Hence, we assessed, by IHC and double IHC staining, the presence of GR1- and CD11b-positive cells at the injury site and compared the levels of these positively staining cells during the phases of the healing process. Rounded, dendritic, and spindle-shaped cells were positive for GR1 and CD11b staining. Almost all of the GR1- and CD11b-double-positive cells were round in shape, but not polymorphonuclear. At PD 3, GR1- and CD11b- single stained cells were widely distributed throughout the injury site (Figure 3A, B), although double-stained cells were seen only rarely (Figure 3C, D, E). At PD 7, GR1-positive cells aggregated around the repaired bone and blood vessels in the transitional area (surrounded by the dotted line) (Figure 3F). However, CD11b-positive cells were more likely to occur in the reactionary area than in the transitional area (surrounded by the dotted line) (Figure 3G). With IHC double staining, cells positive for double staining were seen in the transitional area, proximal to the newly formed bony structure and blood vessels (Figure 3H, I, J).

**Figure 4:**
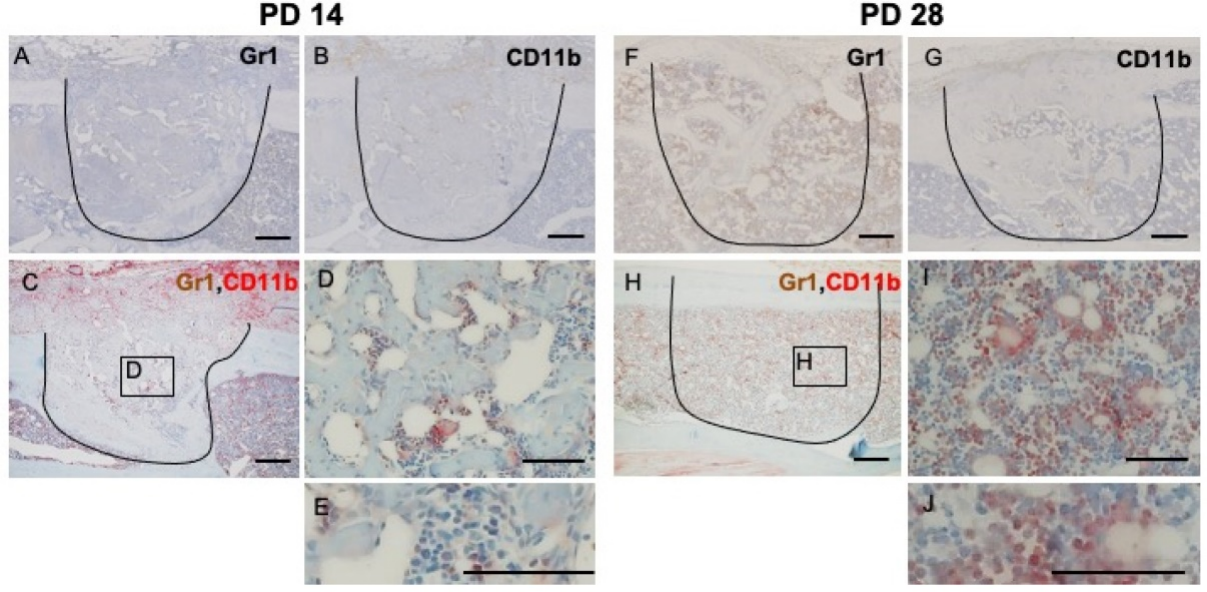
IHC and double IHC characterization and localization of Gr1 and CD11b expression in bony injury site. **At PD14,** the numbers of cells exhibiting staining for Gr1 and CD11b, either alone or together, were significantly decreased (A, B, C, D, E). **At PD28**, the injury site showed complete healing; restored bone marrow cells positive for Gr1 (F) were seen, along with a small number of CD11b-positive cells (G). Some restored bone marrow cells expressed both markers (H, I, J). Scale bars: A, B, C, F, G, and H, 200 µm; D, E, I, and J, 100 µm.

At PD 14, the number of GR1- and CD11b-positive cells decreased dramatically (Figure 4A, B). Negligible numbers of double-stained cells were seen (Figure 4C, D, E). At PD 28, some of the restored bone marrow cells exhibiting staining for GR1 or CD11b (Figure 4F, G). IHC double staining showed that some of the restored bone marrow cells expressed both markers ((Figure 4H, I, J).

### Number of GR1^+^CD11b^+^ MDSCs increases in the transitional area with the shift from reaction to repair phase

**Figure 5:**
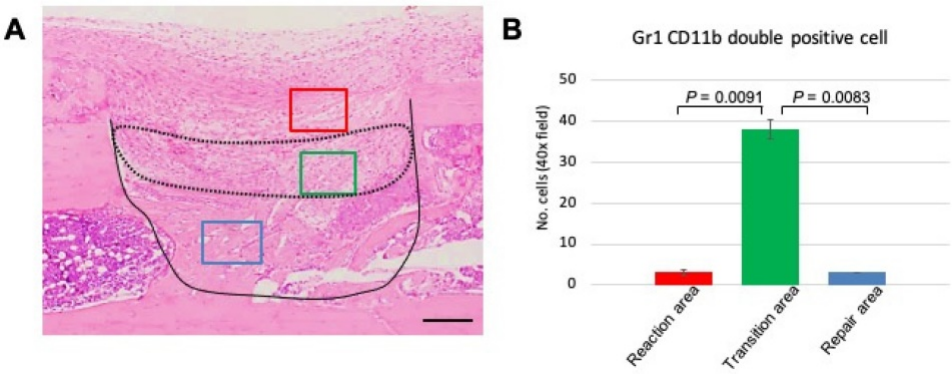
Statistical analysis of GR1, CD11b double-positive cells in the process of bone healing. (A) Demonstration of area division on PD 7 sections; the area surrounded by a dotted line corresponds to the transition area (green box), which is flanked by the reaction area (above; red box) and the repair area (below; blue box). Scale bar: 200 µm. (B) Quantification of GR1-, CD11b double-stained cells in a given field at 400x magnification (n = 3 fields/sample). Statistical significance was assessed using non-paired two-tailed Student's t-tests. Data are presented as mean ± s.d.

The above data demonstrated that GR1 and CD11b expression and localization differed among the various phases of the healing process. The distinct expression profile of MDSCs led us to focus on events at PD 7, a time when we can observe the transition from the reaction to repair phase. Therefore, we performed statistical analysis to assess MDSC emergence at PD 7. First, we divided the healing site into three regions: early reaction, transition, and repair areas (Figure 5A). Three images per sample were used for data analysis. The double-stained cells were counted manually and numbers compared among the three areas. We found that the number of double-stained cells was significantly higher in the transitional area than either of the other two areas (Figure 5B). This finding indicated that the role of MDSCs in the healing process is short-lived, suggesting that this cell type may play a role in establishing an immune balance to facilitate bone repair.

### MDSCs support bone formation

**Figure 6:**
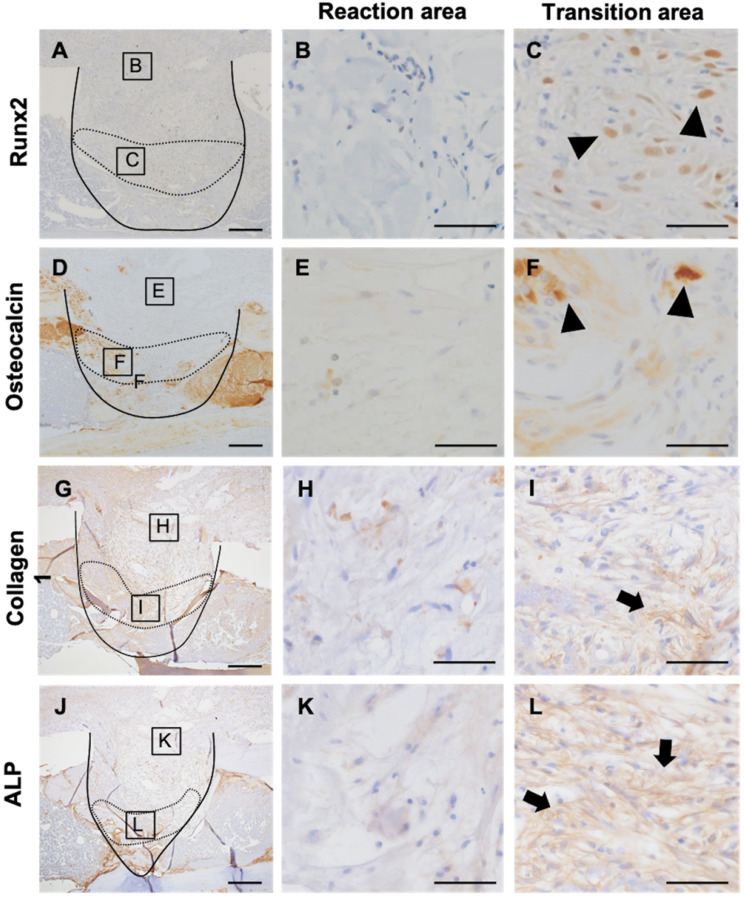
Relation of MDSCs to inflammation and new bone formation. Detection of Runx2, osteocalcin, collagen-1, and ALP expression at PD7 (A-L). Inflammatory area showed no expression of Runx2, osteocalcin, collagen-1, or ALP (B, E, H, K). Runx2- and osteocalcin-expressing cells localized between the area of inflammation and the repair area (*arrowheads*) (C, F). Collagen-1- and ALP-positive spindle-shaped cells localized to the same transition area (*arrow*) (I, L). Scale bars: a and d, 200 µm; b, c, e, and f, 100 µm.

Next, to evaluate the relationship of MDSCs to new bone formation and inflammation, we analyzed osteoblast differentiation using Runx2, a marker of osteoblastic differentiation, and osteocalcin, a marker of mature osteoblasts (Figure 6A, D). To verify osteoblastic differentiation in the transition area, we also analyzed expression of collagen-1 and alkaline phosphatase (ALP). Interestingly, Runx2 staining was observed in the transition area, adjacent to the newly formed bone, but not in the inflammatory area (Figure 6B, C). Osteocalcin-positive cells were observed in the periphery of the newly formed bone (Figure 6E, F). Expression of collagen-1 and ALP localized to the transition area, and cells positive for these markers had round or spindle shapes (Figure 6H, I, K, L). These results suggested that inflammation impedes the differentiation of osteoblasts and MDSCs in the transitional area, further indicating that MDSCs might play a supporting role in new bone formation.

## Discussion

Bone healing is a complex and dynamic process involving many harmonious and coordinated intercellular and molecular interactions 21. During the healing process, the transition from inflammation to repair phase is critical. In the bone injury model used here, the healing phases demonstrate the point of intersection among the various phases: tissues obtained at the PD-3, PD-14, and PD-28 time points showed the reactionary, repair, and remodeling phases, respectively, while tissues obtained at the PD-7 time point showed both the reaction and repair phases. At PD 7, the injury site exhibited three different areas from superficial to deep, corresponding to inflammation, granulation tissue, and repair areas, respectively. This appearance suggested that the middle (granulation) area is a transitional area. Hence, we were able to observe the transition from inflammation to repair at PD 7.

The immune system plays a crucial role in successful healing of bony injuries 22. Neutrophils, macrophages, and dendritic cells stimulate osteoclast formation via the helper T cell (Th 17)-mediated RANKL signaling pathway 23-25. On the other hand, Th1 and Th2 T cells inhibit osteoclast formation 26. Moreover, Treg cells inhibit the differentiation and function of osteoclasts and suppress inflammation 27-29. In recent years, MDSCs have been reported to provide immune suppression by inhibiting the proliferation and function of T cells while stimulating the proliferation of Treg cells 30. These data suggest that MDSCs potentially contribute to bone healing process via immune suppression. Notably, in the present study, GR1- and CD11b double-stained cells localized in the transition area at PD 7. Mature neutrophils and macrophages also express GR1 and CD11b 31,32. However, the cells we identified here as MDSCs do not have the characteristics of mature neutrophils and macrophages. These MDSCs were not observed in the inflammatory area, and the number of these cells decreased significantly at PD 14 and thereafter. These findings suggested that MDSCs accumulate to the highest levels during the transition from the reactionary to repair phase.

Our study showed that Runx2, osteocalcin, collagen-1, and ALP expression level were elevated in cells that localized to the transition area. Runx2 and osteocalcin are markers of osteoblasts and osteoblastic progenitor cells 35. Our data indicated that osteoblast progenitor cells were localized to the transition area, whereupon osteoblast differentiation occurred, as demonstrated by the expression of collagen-1 and ALP by spindle-shaped or round stromal cells 36. Notably, we observed that the localization of GR1- and CD11b double-stained cells was closely related to new bone formation (evidenced by Runx2 expression). We inferred that MDSCs play a role in new bone formation and might be a focal point in balancing the connection between inflammation and bone repair.

## Conclusions

Together, the findings of the present study are first demonstration (to our knowledge) that MDSCs contribute to new bone formation during bone healing. MDSCs may play a role in bone healing by transiently emerging in the transitional period and initiating the repair phase.

## Figures and Tables

**Table 1:** Antibodies used in immunohistochemistry

Antigen targeted by primary antibody	Immunized animal	Antigen retrieval	Dilution	Supplier
GR1	Rat	0.1% Trypsin at 37 °C, 5 min	1:200	Biolegend
CD11b	Rabbit	Microwave heating in 0.01 mol/L citrate buffer (pH 6.0) at 100 °C, 1 min	1:500	abcam
Runx2	Rabbit	Microwave heating in 0.01 mol/L EDTA buffer (pH 9.0) at 100 °C, 1 min	1:1000	abcam
Osteocalcin	Rabbit	Microwave heating in 0.01 mol/L citrate buffer (pH 6.0) at 100 °C, 1 min	1:1000	abcam
Collagen 1	Rabbit	Microwave heating in 0.01 mol/L citrate buffer (pH 6.0) at 100 °C, 1 min	1:500	Cell Signaling
ALP	Rabbit	Microwave heating in 0.01 mol/L citrate buffer (pH 6.0) at 100 °C, 1 min	1:200	TAKARA

GR1 and CD11b double IHC staining.

## Abbreviations

MDSC: myeloid-derived suppressor cells
PD: post-surgical day
IHC: immunohistochemistry
